# The Effect of Bariatric Surgery on Weight Loss and Metabolic Changes in Adults with Obesity

**DOI:** 10.3390/ijerph17155342

**Published:** 2020-07-24

**Authors:** Stanisław Głuszek, Arkadiusz Bociek, Edyta Suliga, Jarosław Matykiewicz, Magdalena Kołomańska, Piotr Bryk, Przemysław Znamirowski, Łukasz Nawacki, Martyna Głuszek-Osuch, Iwona Wawrzycka, Dorota Kozieł

**Affiliations:** 1 The Institute of Medical Sciences, Medical College, Jan Kochanowski University, 25-369 Kielce, Poland; sgluszek@wp.pl (S.G.); jaroslaw.matykiewicz@ujk.edu.pl (J.M.); mkolomanska92@gmail.com (M.K.); piotr.bryk@ujk.edu.pl (P.B.); lukasz.nawacki@ujk.edu.pl (Ł.N.); iwona.wawrzycka@ujk.edu.pl (I.W.); 2 Clinic of General, Oncological and Endocrinological Surgery, Provincial Hospital in Kielce, 25-736 Kielce, Poland; znamirowski79@gmail.com; 3 The Institute of Health Sciences, Medical College, Jan Kochanowski University, 25-369 Kielce, Poland; edyta.suliga@ujk.edu.pl (E.S.); mgluszekosuch@ujk.edu.pl (M.G.-O.); dorota.koziel@ujk.edu.pl (D.K.); 4 Clinic of Oncological Surgery of the Swiętokrzyskie Center of Oncology in Kielce, 25-734 Kielce, Poland

**Keywords:** sleeve gastrectomy (SG), gastric banding (GB), laparoscopic Roux-en-Y gastric bypass (LRYGB), weight loss, metabolic parameters

## Abstract

Methods of treating obesity, such as changes in lifestyle, physical activity, restrictive diets, and psychotherapy, are not sufficient. Currently, it is considered that in the case of patients who meet the eligibility criteria for surgery, the treatment of choice should be bariatric surgery. The aim of this study was to assess the weight loss and metabolic changes in a group of adults with obesity undergoing bariatric surgery. The study involved 163 patients whose body mass index (BMI) exceeded 40 or 35 kg/m^2^, concurrent with at least one metabolic sequelae. In 120 of the cases (74%), sleeve gastrectomy was used; in 35 (21%), gastric banding was used; and in 8 (5%), laparoscopic Roux-en-Y gastric bypass was used. Metabolic parameters such as total cholesterol, LDL-cholesterol (low-density lipoprotein cholesterol), HDL-cholesterol (high-density lipoprotein cholesterol), triglycerides, and glucose were measured preoperatively and postoperatively, as well as the creatinine, creatine kinase (CK-MB), and leptin activity. In patients undergoing bariatric surgery, a significant decrease in excess weight (*p* < 0.001) was observed at all the analyzed time points, compared to the pre-surgery value. Weight loss after surgery was associated with a significant improvement in glycemia (109.6 ± 48.0 vs. 86.6 ± 7.9 mg/dL >24 months after surgery; *p* = 0.003), triglycerides (156.9 ± 79.6 vs. 112.7 ± 44.3 mg/dL >24 months after surgery; *p* = 0.043) and leptin (197.50 ± 257.3 vs. 75.98 ± 117.7 pg/mL 12 months after surgery; *p* = 0.0116) concentration. The results of the research confirm the thesis on the effectiveness of bariatric surgery in reducing excess body weight and improving metabolic parameters in patients with extreme obesity.

## 1. Introduction

One of the biggest challenges for modern medicine is the treatment of eating disorders and metabolic disorders, in particular, obesity, as well as the prevention of their complications. In 2016, among the world’s population, 39% of adults (39% men, 40% women) were overweight (body mass index (BMI) of ≥25 kg/m^2^) and 13% were obese (11% men, 15% women) (BMI of ≥30 kg/m^2^). Additionally, in Poland 59% of adults (63% men, 55% women) were overweight and 25% were obese (24% men, 27% women). In Poland, among school students aged around 11, the incidence of overweight or obesity was 36% among boys and 23% among girls [1,2]. Despite the actions taken among both children and adults, a progressive increase in average body weight has been observed [1,2,3,4,5].

However, the biggest problem associated with obesity is not the excess of adipose tissue itself, but the metabolic disorders and complications resulting from the disease, including the increased risk of premature death [4,6]. Metabolic syndrome (MetS) is particularly related to excess weight. Although MetS may be present even in people with a high normal BMI (BMI in range of 23–25 kg/m^2^), especially those with low physical activity or unhealthy nutritional patterns [7,8,9,10], the highest probability of the occurrence of MetS and other complications is associated with obesity (BMI of ≥30 kg/m^2^); these include type 2 diabetes, hypertension, cardiovascular disease (with an increased risk of myocardial infarction and stroke), obstructive sleep apnea, lipid disorders, osteoarthritis, and some cancers (endometrial, breast, ovarian, prostate, esophageal, hepatic, gallbladder, kidney, and colon) [1,2,3].

The existing conservative methods of treatment, including changes in lifestyle, physical activity, restrictive diets, and psychotherapy, are not very effective, resulting in only up to 10% of the desired weight loss [11]. Maintaining body weight is a much more difficult problem, and reduces the effectiveness of these methods of therapy [11,12,13]. The pharmacological methods of treatment of obesity are lipostatins (orlistat); drugs restricting appetite (bupropion, naltrexone); and others, such as lorcaserin, phentermine-topiramate, or liraglutide. All of these medicines were effective in previous studies, with a loss of weight of about 5% after 52 weeks of therapy [14].

Taking the above into consideration, at the current level of medical knowledge bariatric surgery is the most effective treatment for obesity and is now considered to be the treatment of choice in patients who meet the eligibility criteria for surgery [3,15,16]. The main indications for surgery in adults are a BMI ≥ 40 kg/m^2^ or a BMI ≥ 35 kg/m^2^, with the simultaneous occurrence of at least one disease caused by obesity. In children and adolescents, surgery should be considered with a BMI ≥ 40 kg/m^2^ and one obesity-related disease, or with a BMI ≥ 35 kg/m^2^ with severe comorbidities resulting from the excess weight [3].

Bariatric procedures include, among others, sleeve gastrectomy (SG); laparoscopic Roux-en-Y gastric bypass (LRYGB); gastric banding (GB); and the gastric balloon, which is mainly used in preparation for other bariatric operations [17].

However, there are still insufficient data to clearly indicate which type of surgery is most effective in a given group of patients. The results of research on postoperative metabolic effects, such as changes in the lipidogram, are also ambiguous. Spivak et al. [18] found that different types of bariatric surgery had different effects on dyslipidemia, regardless of weight loss. LRYGB was associated with the greatest reduction in the concentration of total cholesterol and Low-Density Lipoproteins (LDL-cholesterol) in plasma, while SG influenced the concentration of High-Density Lipoproteins (HDL-cholesterol) to the greatest extent. Szczuko et al. [19] did not observe statistically significant differences in glucose concentrations after the LRYGB and SG procedures. The highest differences were observed for triglycerides and all cholesterol fractions, which decreased after LRYGB but increased in the first months after SG surgery. Carswell et al. [20] observed that triglycerides decreased 3 months after surgery, but unlike other studies they found that HDL increased from 1 year after surgery.

The aim of the study was to assess the loss of weight and metabolic changes in a group of adults with obesity undergoing bariatric surgery.

## 2. Materials and Methods

Initially, 184 patients eligible for bariatric surgery were enrolled—i.e., those whose BMI exceeded 40 kg/m^2^ or 35 kg/m^2^ with at least one obesity complication. In the study patients were included after the following surgeries: sleeve gastrectomy (SG), gastric banding (GB), and laparoscopic Roux-en-Y gastric bypass (LRYGB). The exclusion criteria included lack of consent for surgery; general health according to World Health Organization (WHO) performance status 2 (presence of disease symptoms, significantly limited activity, ability to perform daily activities, lack of ability to perform work, the need to stay in bed less than 50% of the day); above all, a negative opinion on the psychological preparation of the patient, stating the inability to change patient lifestyle after surgery, and being below 18 years of age or above 65. At the final stage, 163 patients qualified for the study.

In the patients included in the study, metabolic parameters such as total cholesterol, LDL, HDL, triglycerides, and glucose were measured preoperatively and postoperatively, as well as the muscle breakdown parameters creatinine and creatine kinase (CK-MB) in the preoperative period and in the early stages after the surgery (3–5 days), and leptin activity (double examination—before and about 12 months after the procedure). In addition, the concentration of lipids and glucose was assessed during follow-up visits at the following time points after surgery: 1 month, 3 months, 6 months, 12 months, 24 months, and after more than 24 months. After an extensive surgery, the patient may develop rhabdomyolysis and following acute kidney injury, thus creatinine kinase and creatinine measuring was performed [21,22]. Almost none of the patients suffered from diabetes before treatment. Serum fasting glucose level is the main parameter to diagnose conditions such as diabetes and impaired fasting glucose. According to some researches, the usefulness of HbA1c for the diagnosis of impaired fasting glucose and diabetes in patients aged <30 years remains to be determined due to discrepancies between the results of glucose- and HbA1c-based tests. This was the main reason to use the serum fasting glucose instead of HBa1c [23]. According to some studies, improvements in leptin and ghrelin levels following bariatric surgery appear to contribute to postoperative cognitive benefits. These gains may involve multiple mechanisms, such as reduced inflammation and improved glycemic control [24].

The BMI, ideal body weight, and excess weight were calculated based on the measured height and body weight. Ideal body weight (IBW) is the patient’s weight calculated using the following formula [20]:

Men: IBW = 50 kg + ((height − 150 cm) 0.7 kg/cm),
Women: IBW = 50 kg + ((height − 150 cm) 0.6 kg/cm).

The observed changes in BMI, excess weight, or metabolic parameters could not be compared by age and sex due to the limited probe size at each time point.

In the postoperative period, for the first two weeks (from the third day onward) the patients were nourished with a liquid diet complemented by liquids in a summary quantity of 1500 mL per day. Further, the limitation of calories was established at the level of 1200–1500 kcal per day. Then, it was gradually adjusted to the individual demand of each patient. The diet was complemented by supplementation with vitamins and minerals, especially B_1_, B_6_, D_3_, and iron. The intake of these supplements was recommended depending on the results of laboratory analyzes to achieve a laboratorial normal range and clinical balance.

For the statistical description of quantitative features, we used the arithmetic means, standard deviations, medians, quartiles, and ranges of values (minimum and maximum). The distributions of qualitative features were described by way of frequency and percentages. The frequencies were compared using the chi-square test or Fisher’s exact test. The normality of distributions was checked using the Shapiro Wilk test. For data compliance with the normal distribution, the Student’s *t*-test was used to compare distributions (for independent samples or for pair-related samples). In the absence of the normality of distributions, the Mann Whitney *U* test was used for the independent samples, and the Wilcoxon signed-rank test was used for two related samples. The correlations between pairs of quantitative variables were evaluated using the Spearman rank correlation coefficient. All the statistical tests performed were bilateral, and *p* < 0.05 was accepted as the statistical significance criterion. The STATISTICA program was used in the calculations (TIBCO Software Inc. (2017), Statistica (data analysis software system) version 13. http://statistica.io, Palo Alto, Santa Clara, CA, USA).

The study was approved by the Committee on Bioethics at the Faculty of Health Sciences, Jan Kochanowski University in Kielce, Poland, on 26 March 2018 (number of approval: 24/2018). All the participants gave written consent to participate in the study.

## 3. Results

### 3.1. Characteristics of the Study Group

The analysis included patients who underwent surgery and had at least one check-up (BMI, biochemical tests) in the postoperative period. The study involved 163 patients, including 136 women (83%) and 27 men (17%), of whom 75 female (46%) and 14 male (9%) patients were under 40 years old and the remaining 61 women (37%) and 13 men (8%) were aged 40 or above. Forty-four subjects (27%) were free from comorbidities. Among the 119 patients (73%) in whom comorbidities were present, the most common were the following: hypertension (67 cases, 40%), diabetes (40%), obstructive sleep apnea (8%), and ischemic heart disease (5%). There was no anastomotic leak after SG or after LRYGB. In the early postoperative period, one case of gastrointestinal bleeding after LRYGB and one case of bleeding into the peritoneal cavity after SG were found, with a slight intensity (bleeding from the drain). The patients did not require surgical procedures, though the patient after LRYGB required the substitution of red blood cell concentrate.

The other parameters of the test group before bariatric surgery are presented in Table 1, taking into account the division into subgroups according to sex. It is shown that the men in the analyzed group were statistically significantly (*p* < 0.05) older; were taller; and had a higher initial weight, BMI, ideal body weight, and initial excess weight.

Among the patients who underwent surgery, in 120 (74%) SG was used; in 35 (21%), GB was used; and in 8 (5%), LRYGB was used. Almost one-third of the patients (51 observed subjects) were examined only on the day of the surgery and did not report for follow-up visits, so a BMI assessment in the follow-up period was not available in these cases. The total number of patients whose BMI was examined at least twice (including once before the procedure) was 112 (67%).

### 3.2. Analysis of Weight Loss

A significant decrease in excess weight in relation to the pre-treatment value was observed in all the analyzed time points (*p* < 0.001). The final average value of weight decrease (after over 24 months) was 30.3 kg. Details on the loss of excess weight are shown in Table 2. However, no significant differences were found in relation to sex or age (groups of ≤40 and >40 years were compared) (data not shown).

Significant differences were also observed by comparing time points 6 and 12 (6 and 12 months after surgery) and 24 and 12 (*p* < 0.001). Moreover, for the pairs of points 1 and 0 (1 month after surgery and before surgery), 6 and 0, 12 and 0, 24 and 0, >24 and 0, and 6 and 12, a positive correlation of weight loss was observed (*p* < 0.001, adjusted using the false discovery rate method).

**Figure 1:**
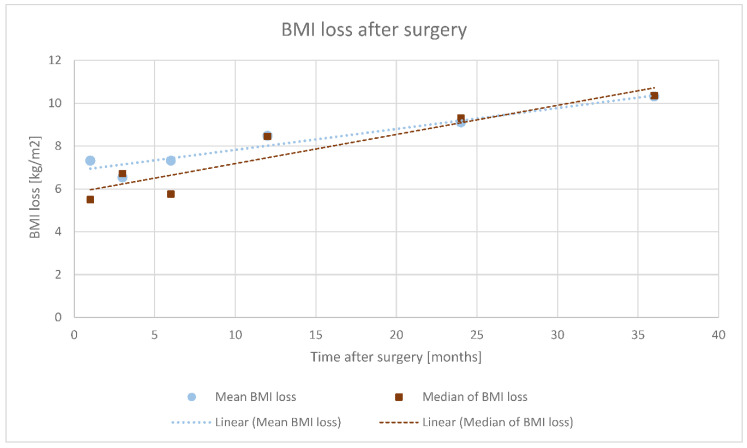
Body mass index (BMI) changes at individual follow-up time points in relation to the presurgery BMI measurements.

Figure 1 shows the mean and median values of the BMI changes relative to pre-operative BMI values. Comparing the time points 6 and 0, 12 and 6, and >24 and 12, it was found that the more time that had elapsed since the surgery, the greater the BMI loss was (*p* < 0.001 for each comparison)—i.e., the highest loss in BMI was recorded in the group with more than 24 months of observation time (average of 10.32 kg/m^2^ BMI loss after over 24 months compared to 8.48 kg/m^2^ after 12 months and 7.32 kg/m^2^ after 6 months).

During follow-up, in 18 patients (11%) a ”rebound effect” was also observed, which is a re-increase in the patient’s weight during the follow-up visits. Most often, the “rebound effect” was observed 24 months after the surgery (21% of patients were examined at this time point). In 7% of the patients examined after 6 months and 13% examined after 12 months, a transient increase in weight was observed. However, in most of these patients (5% after 6 months and 9% after 12 months) their weight decreased again in following time points. The “rebound effect” was also observed in patients after 24 months (13%), but it is unknown whether their mass would stay increased or decrease because they did not undergo any further control examination.

**Figure 2:**
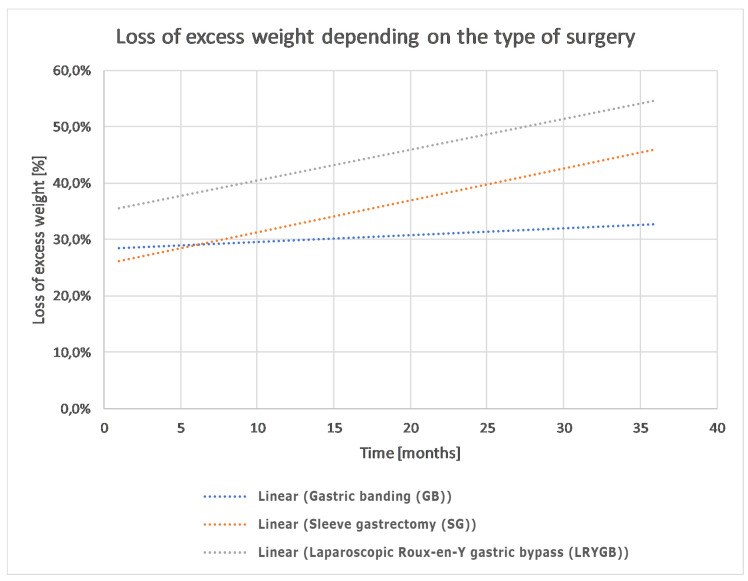
Trend lines of excess weight loss (%) in relation to excess weight before surgery, depending on the type of procedure.

The loss of excess weight depending on the type of surgery (SG vs. GB vs. LRYGB) is shown in Figure 2. The analysis showed that the BMI loss did not differ significantly (*p* > 0.05) depending on the type of surgery, which might be due to the lack of power of the performed test caused by the probe size limitation.

### 3.3. Analysis of Metabolic Parameters

Before surgery glucose (*n* = 120), the triglycerides (*n* = 83) and total cholesterol (*n* = 75) were measured. The number of patients with measured metabolic parameters measured is presented in Table 3. Changes in the glucose levels showed a steady downward trend during the year after the surgery and showed statistical significance (*p* = 0.003) (Table 4). The mean glucose concentration before the procedure was 110.45 mg/dL, and that one year after the procedure was 89.88 mg/dL. The percentage of patients with normal fasting glycemia increased from 40% before surgery to 67% one year after surgery. The decrease in glucose concentration was not correlated with the loss of weight. An analysis of the changes in cholesterol levels showed significant fluctuations of this parameter within one year after the procedure; however, these changes did not correlate with weight loss. Due to the above, it was not possible to determine the trend of changes after surgery. The total percentage of patients with cholesterol levels of >190 mg/dL remained constant and amounted to 39%. The other parameters of the lipidogram (total cholesterol, LDL, HDL) also showed no significant changes during the follow-up period for individual patients. However, significant differences were found for mean triglyceride concentrations between the time points (before the surgery and after 1, 6, 12, or more months) (*p* = 0.043).

There were no significant changes in the serum creatinine before or after the procedure (Table 5). These values were within the reference range for over 90% of patients. However, the analysis showed a significant decrease in leptin concentration when comparing the concentrations before and 12 months after surgery (*p* = 0.0116).

## 4. Discussion

Obesity as a disease is associated with a significant increase in mortality and many health threats, including type 2 diabetes, hypertension, dyslipidemia, coronary heart disease, development of cancer, and osteoarticular disorders. The higher the body mass index (BMI), the higher the risk of morbidity and mortality [25]. Randomized studies have shown that weight loss through lifestyle or pharmacological treatment reduces morbidity by reducing risk factors for cardiovascular disease (CVD) [26], although its efficacy is less than that of surgical treatment. For people who are unable to reduce their body weight by means of behavioral therapy and pharmacological treatment, surgical treatment should be considered. However, even then appropriate behavioral therapy with adequate dietary recommendations and physical activity adapted to the patient’s ability is necessary [27,28].

The slightly lower loss of weight and BMI 3, 6, and 12 months after bariatric surgery in our examined group in comparison with the outcomes of other authors, might be result of, firstly, the lower initial BMI of our patient group (X ± SD) = 44.5 ± 6.8 kg/m^2^; Me = 43.4 kg/m^2^ (40.2–46.3) vs. X = 45.91 kg/m^2^ (min–max 41.40–50.11) [29] and Me = 51.6 kg/m^2^ (35.9–72.0) [30]. Secondly, it might be a result of the fact, that in 21% of our patients, GB surgery was performed. The difference in loss of weight depending on the surgery method was statistically insignificant (most probably due to the limitation of size); however, the loss of weight observed after this type of surgery was noticeably lower. In the cited studies [29,30], all the patients underwent SG. From a long-term perspective (>24 months of follow-up), our results did not differ significantly from others. The average BMI loss after 24 months was 10.3 kg/m^2^, while the average percentage of excess weight loss was 50.5%. In a study of Kowalewski et al., the analogous loss of BMI was 12.1 kg/m^2^, while the average percentage excess weight loss was 51.1% [30].

The results of our own research confirm favorable metabolic changes in the course of bariatric treatment, a significant improvement in glycemia and triglycerides, and a tendency to normalize HDL cholesterol. In the 12 months after surgery, the participants of the follow up reached lower glucose concentrations (89.9 vs. 98.0 mg/dL), similar to how the concentrations of total cholesterol, LDL, and TG behaved, while a lower HDL (42.6 vs. 55.0 mg/dL) was observed compared to that of the patients in the study by Wojciak et al. [29]. Our patients reached a significant improvement in the concentration of HDL (61.9 mg/dL) a bit later, more than 12 months after surgery. The significant decrease in leptin concentration in the group of patients we studied is consistent with the observations of other authors, who indicated that rapid weight loss in morbidly subjects with obesity undergoing bariatric surgery leads to significant changes in the concentration of some adipokines and hormones that control the appetite and energy processes in peripheral blood [31]. In diabetic patients, weight loss following surgical treatment makes possible the withdrawal of antidiabetic agents or a reduction in doses. In studies by other authors [32], diabetes prevention programs significantly reduced the rate of progression from impaired glucose tolerance to diabetes over three years in participants randomly assigned to intensive lifestyle modification focusing on weight loss [14]. The effectiveness of intensive lifestyle modification in the prevention of diabetes persisted for 15 years, but over time it weakened [32]. Definitely improved diabetes control results were obtained after surgical treatment by performing laparoscopic Roux-en-Y gastric bypass (LRYGB), and to a lesser extent when using sleeve gastrectomy [29]. After bariatric treatment, there is a sustained reduction in risk factors for cardiovascular diseases (CVD) [33,34]. Similar observations were made in the individuals in the group we have studied. In some patients, the doses of antidiabetic agents have been reduced, and some individuals no longer have to take any antidiabetic agents. Such observations concern those patients in whom the effect of weight loss was significant (over 50% excess weight loss). In most cases, the lack of significant weight loss or the maintenance of intensive lifestyle modification weakens the “antidiabetic” effect and does not significantly improve other elements of quality of life, such as ailments related to the condition of the musculoskeletal system. The duration of diabetes and obesity is also important in the prevention of diabetes or in predicting the remission of diabetes after bariatric treatment. The earlier the bariatric treatment, the greater the chance of avoiding the development of diabetes or reducing the use of drugs (due to preserved endocrine capacity of the pancreas). Similar results were obtained in studies comparing the effects of laparoscopic Roux-en-Y gastric bypass (LRYGB) in adolescent and adult patients (BMI values of 54 kg/m^2^ and 51 kg/m^2^). Diabetes remission was higher in adolescents (86%) compared to adults (53%), as was hypertension (68% vs. 41%). Within 5 years of surgery, 3 in 161 patients (1.9%) and 7/396 (1.8%) adults died [35].

A number of observational studies also confirmed a reduction in mortality along with weight loss [28,31]. Arteburn et al. [36] showed that among surgical patients (*n* = 2500 mean age, 52; mean BMI 47 kg/m^2^) and patients in a control group (*n* = 7462; average age, 53; average BMI 46 kg/m^2^), there were 263 deaths in the surgery group (mean follow-up period, 6.9 years) and 1277 deaths in the control group (mean follow-up period, 6.6 years). In the whole group of patients, complications within 30 days were found in 8.4%. For patients aged 50 to 54 years, 55 to 59 years, and ≥60 years, this risk increased significantly to 9.8%, 10.0%, and 10.2%, respectively. The indicators of specific surgical complications, such as an anastomotic leak, bleeding, and deep infections/abscesses, were significantly increased by 14% to 41% in patients aged 50 to 54 years with a slight additional, though insignificant, risk increase in older patients.

Patients with obesity should be treated in a particularly individual way in terms of the choice of procedure. An assessment of their general health, cardiovascular fitness, respiration, excretory system health (kidney function), and neurological status are key elements in deciding upon a treatment. The risk of complications exceeding the benefits of surgical treatment and the risk of excessive side effects of pharmacological treatment indicate the need to abandon such treatments. However, for a vast majority of patients with class I to class III obesity, after unsuccessful conservative treatments surgical treatment already has a documented value. In most cases, weight loss is associated with an improved quality of life (improvement of mobility, resolution of sleep apnea), a reduction in the components of metabolic syndrome and glycemic and lipidogram disorders, and a lower risk of cardiovascular illnesses and death.

A limitation of this study was the relatively small number of participants and the time of follow-up. Despite the still-rising percentage of people suffering from morbid obesity (BMI of ≥40 kg/m^2^) in Poland, compared to that in other countries, this percentage is still fairly small (only 1.3% of men and 1.8% of women) [37]. Another limitation was the relatively small number of patients in whom the LRYGB was performed, which made it difficult to compare the results of the surgery methods.

## 5. Conclusions

In patients undergoing bariatric surgery, a significant decrease in excess weight was observed at all analyzed points in time compared to the pre-operative values. The highest weight loss was noted more than 24 months after the procedure. Bariatric treatment was associated with a significant improvement in glycemia, triglycerides, and leptin.

The results of this study confirm the thesis on the effectiveness of bariatric surgery in reducing excess body weight and improving metabolic parameters in patients with extreme obesity. It is necessary to conduct further, long-term observations in order to identify the most effective yet safe methods of bariatric treatment.

## Figures and Tables

**Table 1:** Mean values of the parameters of the study group before bariatric surgery.

Parameters	Total (*n* = 163)		Women (*n* = 136) X ± SD	Men (*n* = 27) X ± SD	*p*-Value
	X ± SD	Me (Q1–Q3)			
Age	39.6 ± 10.6	40.0 (31.0–46.0)	38.4 ± 10.0	43.4 ± 11.5	0.0071
Height (cm)	168.2 ± 8.5	167.0 (164–172)	166.3 ± 8.4	175.4 ± 9.5	<0.0001
Initial weight (kg)	123.6 ± 21.5	120.0 (109–134)	120.9 ± 19.4	148.1 ± 25.0	<0.0001
Initial BMI (kg/m^2^)	44.5 ± 6.8	43.4 (40.2–46.3)	44.0 ± 9.2	48.1 ± 8.3	0.0003
Initial ideal body weight (kg)	61.3 ± 5.8	60.2 (58.4–64.3)	59.8 ± 5.0	67.8 ± 6.7	<0.0001
Initial excess weight (kg)	62.3 ± 18.5	59.8 (49.8–72.0)	61.2 ± 18.6	80.15 ± 23.4	<0.0001

X: arithmetic mean; SD: standard deviation; Me: median; Q1: first quartile; Q3: third quartile; BMI: body mass index.

**Table 2:** Excess weight loss (X%) compared to the pre-operative excess weight value (100%) at particular points in time during the follow up.

Time after Procedure (Months)	*N*	Excess Weight Loss			
		X ± SD (kg)	X (%)	Me (kg)	Me (%)
1	37	18.5 ± 16.3	27.9	16.5	24.9
3	17	18.3 ± 10.4	28.4	17.5	26.8
6	42	19.4 ± 13.4	31.4	17.0	28.2
12	47	23.9 ± 11.0	40.4	23.0	39.0
24	39	25.1 ± 13.7	44.4	24.5	42.8
>24	19	30.3 ± 19.2	50.5	31.0	56.5

X: arithmetic mean; SD: standard deviation; Me: median.

**Table 3:** Number of patients with metabolic parameters measured (1, 6, 12, and >12 months after surgery).

Biochemical Parameters	Time (Months) After Surgery			
	1	6	12	>12
Glucose	4	9	8	8
Triglyceride	23	15	14	10
Total cholesterol	28	23	19	12
LDL	18	5	6	6
HDL	17	6	5	8

LDL: low-density lipoprotein; HDL: high-density lipoprotein.

**Table 4:** Mean concentrations of biochemical parameters measured before the procedure and during the follow-up period (mg/dL).

Biochemical Parameters (mg/dL)	Before Surgery (X ± SD)	Time (Months) After Surgery				*p*-Value
		1 (X ± SD)	6 (X ± SD)	12 (X ± SD)	>12 (X ± SD)	
Glucose	109.6 ± 48.0	104 ± 8.1	91.3 ± 14.3	89.9 ± 18.3	86.6 ± 7.9	0.003
Triglyceride	156.9 ± 79.6	137.7 ± 55.2	126.5 ± 34.3	111.6 ± 43.7	112.7 ± 44.3	0.043
Total cholesterol	198.4 ± 47.8	188.7 ± 36.7	165.2 ± 82.5	186.9 ± 35.3	191.7 ± 42.7	0.180
LDL	-	122.7 ± 40.8	137 ± 32.6	112 ± 18.8	131 ± 36.6	0.261
HDL	-	41.2 ± 12.0	41.7 ± 7.8	42.6 ± 6.8	61.9 ± 23.9	0.084

X: arithmetic mean; SD: standard deviation; LDL: low-density lipoprotein; HDL: high-density lipoprotein.

**Table 5:** Mean concentrations of the biochemical parameters associated with surgery measured before surgery and 12 months after surgery.

Biochemical Parameters	Concentration before Surgery		Concentration 12 Months after Surgery		*p*-Value
	X ± SD	Me	X ± SD	Me	
Creatinine (mg/dL)	4.87 ± 18.3	0.88	2.93 ± 13.7	0.86	0.3912
CK-MB (mg/dL)	15.61 ± 15.8	14.0	13.97 ± 3.6	14.0	0.6056
CPK (mg/dL)	156.1 ± 134.5	113.0	192.24 ± 112.1	155.0	0.1240
Leptin (pg/mL)	197.50 ± 257.3	50.0	75.98 ± 117.7	39.8	0.0116

CK-MB: creatine kinase fraction of the heart; CPK: phosphocreatine kinase; X: arithmetic mean; SD: standard deviation; Me: median.
